# Models of cartilage repair with autologous mesenchymal stem cells seeded on scaffolds: a systematic narrative review

**DOI:** 10.3389/fbioe.2026.1762579

**Published:** 2026-03-03

**Authors:** Mikołaj Wróbel, Hubert Rytel, Igor Jaszczyszyn, Maciej Maj, Jacek Malejczyk, Izabela Róża Janiuk

**Affiliations:** 1 Department of Orthopaedics and Traumatology, Carolina Medical Center, Warsaw, Poland; 2 Department of Histology and Embryology, Center of Biostructure Research, Medical University of Warsaw, Warsaw, Poland; 3 Faculty of Medical and Health Sciences, Institute of Health Sciences, University of Siedlce, Siedlce, Poland

**Keywords:** animal models, articular cartilage, autologous transplantation, mesenchymal stem cells (MSCs), scaffolds, seeded scaffolds, tissue engineering, translational models

## Abstract

Focal post-traumatic cartilage lesions frequently progress to early osteoarthritis, highlighting the limited regenerative capacity of adult articular cartilage. Compared to native tissue, conventional surgical interventions often produce fibrocartilage with inferior biomechanical properties, representing a persistent therapeutic challenge. This review assessed preclinical studies exploring cartilage repair strategies using autologous mesenchymal stem cells (MSCs) in animal models. MSCs therapies demonstrated superior cartilage regeneration, matrix organization, and integration into the surrounding tissue compared to the control groups. The most efficient source was found to be bone marrow - derived mesenchymal stem cells (BM-MSCs) combined with biodegradable scaffolds, suggesting their potential in tissue engineering applications. Despite methodological heterogeneity across studies - including variations in stem cells sources, implant types, and deliver strategies - cumulative evidence strongly supports the regenerative potential of autologous MSCs for cartilage repair. Current research identifies key knowledge gaps, including the absence of standardized experimental protocols and limited insight into the mechanisms of tissue remodeling and maturation. Collectively, these gaps limit direct clinical translation, highlighting the need for further, standardized studies in large animal models with long-term follow-up (>2 years) to assess integration, functional maturation, and the full regenerative potential of the repair tissue.

## 1 Introduction

Osteoarthritis (OA) is a highly prevalent degenerative joint disease, most commonly affecting the knee. OA is a leading cause of pain and disability worldwide, creating significant socioeconomic and healthcare challenges (Alkady et al., 2023). While age-related OA is a major contributor to pain or dysfunction in the elderly population (Richmond et al., 2013; Bornes et al., 2014), post-traumatic cartilage defects, frequently sport-related and accompanied by ligament tears, pose a major risk for the early onset of OA in young, active individuals (Steinwachs et al., 2012).

Mechanical repair strategies, including marrow stimulation, microfractures, and autologous osteochondral transplant (OAT), can alleviate symptoms and restore surface continuity (Le et al., 2020). Nevertheless, their long-term efficacy is limited, as these approaches do not reliably regenerate durable, native hyaline cartilage, resulting in repair tissue with inferior mechanical properties and suboptimal integration with the surrounding native matrix (Bornes et al., 2014). Owing to these limitations, therapies utilizing mesenchymal stem cells (MSCs), especially delivered on a biomaterial scaffold, have gained increasing interest for promoting targeted regeneration of hyaline-like cartilage and restoration of the osteochondral unit (Bornes et al., 2014).

**FIGURE 1:**
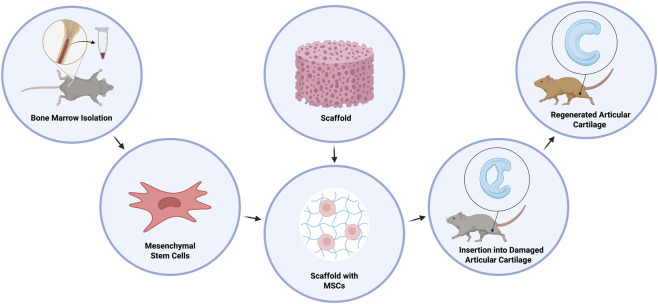
Schematic representation of mesenchymal stem cells (MSCs) based therapy for articular cartilage repair. Bone marrow is aspirated, then MSCs are isolated and expanded *in vitro*. MSCs are then combined with a biocompatible scaffold and implanted into the site of damaged articular cartilage (Rytel, 2025b).

Among different MSCs delivery formats, implantation of MSCs on a scaffold directly into the joint defect, as shown on Figure 1, may provide better spatial control and enable the assessment of structural endpoints, in contrast to intra-articular injection, where both precise localization and standardized grading remain challenging (Le et al., 2020). This approach is expected to meet several key conditions for successful treatment. Firstly, specific scaffold choice and its fixation are of prime importance, as the implant must conform to and effectively seal the defect. Secondly, it should be biodegradable, allowing for gradual absorption, and biocompatible to prevent potential inflammatory reactions or rejections as a foreign body. Finally, it should create a microenvironment that supports MSC differentiation into chondrogenic lineages while simultaneously ensuring stable integration with the adjacent cartilage and subchondral bone (Liang et al., 2023; Jelodari et al., 2022). Cell delivery in such procedures can be done either *ex vivo*, before placing the scaffold into the defect, or *in vivo*, where the scaffold is first placed and stabilized in the damaged area, followed by the injection of cultured cells or concentrate (Le et al., 2020).

Preclinical studies based on varied species show that MSCs scaffold constructs may improve integration and matrix quality compared to acellular scaffolds (Lee et al., 2023). Large and small animal models describe integration at the cartilage margin as a key determinant of the procedure success (Lee et al., 2023; Araki et al., 2015; Pot et al., 2017). Despite limited research on mechanical characterization, indentation and nanoindentation analyses in selected animal models have demonstrated that combining MSCs with engineered scaffolds can enhance tissue properties towards native-like cartilage (Huang et al., 2018; Getgood et al., 2012; ShimomuraKazunori et al., 2014-03).

Clinically, procedures that seed MSCs onto scaffolds have shown sustained improvements in patient reported outcomes, with supportive magnetic resonance imaging (MRI) and limited second look histology. Although clear mechanical evidence in humans remains unsatisfactory, studies show that mechanical and histological restoration could be model and time-point evaluation dependent (Lee et al., 2023; Getgood et al., 2012; Krych et al., 2016).

Despite the large number of experimental studies, there is a lack of standardization regarding several critical variables, including the minimum effective number of cells introduced into the defect, their survival time, the optimal source of their extraction, cultivation time, and the number of cell passages before the implantation. In addition, diverse types of scaffolds with varying building biological properties have been implemented. These variables substantially influence the efficacy of potential therapies, complicating definitive conclusions.

This review provides a comprehensive synthesis of *in vivo* preclinical studies focusing on the use of MSCs seeded on different scaffolds, excluding injection-only or non-autologous strategies, in cartilage repair. This work integrates histological, molecular, imaging, and biomechanical findings to identify limitations in outcome assessment and synthesize priorities for standardization.

## 2 Materials and methods

This study was conducted as a systematic narrative review, integrating the methodological rigor of a systematic review with the interpretive depth of a narrative synthesis. A comprehensive literature search was carried out across multiple databases in accordance with PRISMA guidelines, using predefined keywords and inclusion/exclusion criteria. Eligible studies were screened, critically appraised, and synthesized narratively to provide an overview of current knowledge, highlight key biological and mechanistic insights, and identify gaps for future research.

### 2.1 Searching criteria

A thorough literature search was conducted from inception to August 2023 and was updated on 22 January 2026 to include studies published up to the search date, using the following databases: PubMed, Scopus, Web of Science, and EMBASE. The specific details of search methodology are provided in the Supplementary Material S1. All studies were uploaded to Endnote (Team and T.E., 2013) where duplicates were deleted. Subsequently, studies were uploaded to the Rayyan website (Ouzzani et al., 2016), where they were checked again for duplicates, and if any occurred, they were deleted. Ig.J. and M.W independently, with the blind mode on, screened articles titles and abstracts before proceeding with a full-text screen. If there were any disagreements without resolution, a third reviewer Iz.J. was consulted.

### 2.2 Inclusion and exclusion criteria

Studies were included if they met following conditions:

1. Population (preclinical, non-human): *In vivo* animal models with focal articular cartilage or osteochondral defects (any mammalian species; any joint).
2. Intervention: Autologous mesenchymal stromal/stem cells (MSCs) delivered on/within a scaffold or hydrogel (e.g., collagen, PLGA, HA, PCL, PVA/CS, silk-nanoCaP, OPF, fibrin, etc.). Genetic modification of MSCs (e.g., CDMP1) is allowed.
3. Autologous definition: Donor and recipient are the same animal.
4. Scaffold definition: Any 3D biomaterial (including hydrogels) intended to retain/deliver cells at the defect and support tissue formation.
5. Comparators: Acellular scaffolds, empty defects, or alternative cell types (e.g., chondrocytes) permitted as control/comparator arms.
6. Outcomes (must report ≥1): Histology/histochemistry/IHC (e.g., Safranin O, collagen I/II/X), quantitative matrix metrics (GAG/DNA), gene expression (e.g., COL2A1, ACAN, SOX9), macroscopic scores (ICRS/O’Driscoll), imaging (MRI), or biomechanical readouts.

Studies were excluded if they met any of the following conditions:

1. Human studies (any phase clinical studies or case reports).
2. Non-autologous cells as the intervention (allogeneic/xenogeneic MSCs) unless used only as comparators.
3. No scaffold delivery: Cell injections or microfracture/BCP alone without a cell-laden scaffold.
4. Scaffold-only studies without any cell-seeded arm (unless serving exclusively as comparators to an included MSC-scaffold arm within the same paper).
5. Non-MSC therapies as the intervention (e.g., chondrocytes, iPSCs) unless comparator-only. Secretome-only approaches: MSC-conditioned media, extracellular vesicles, or exosomes without implanted MSCs.
6. In vitro-only studies without any *in vivo* implantation arm.
7. Irrelevant indications (e.g., intervertebral disc, nasal septum) unless articular cartilage/osteochondral defect data are separable and reported.
8. Language other than English.

### 2.3 Data extraction and synthesis

Data was extracted by the M.W., Ig.J., H.R., Iz.J. independently in the created Excel datasheet. Following parameters were extracted:

- Study features and demographic attributes: country of the study, purpose of the work, experimental methods, species studied (including information about sex, age and mean weight).
- Treatment information: place of cell isolation, type of cells, MSCs density, cells subculture, treatment type, transplant category and scaffold characteristic.

**FIGURE 2:**
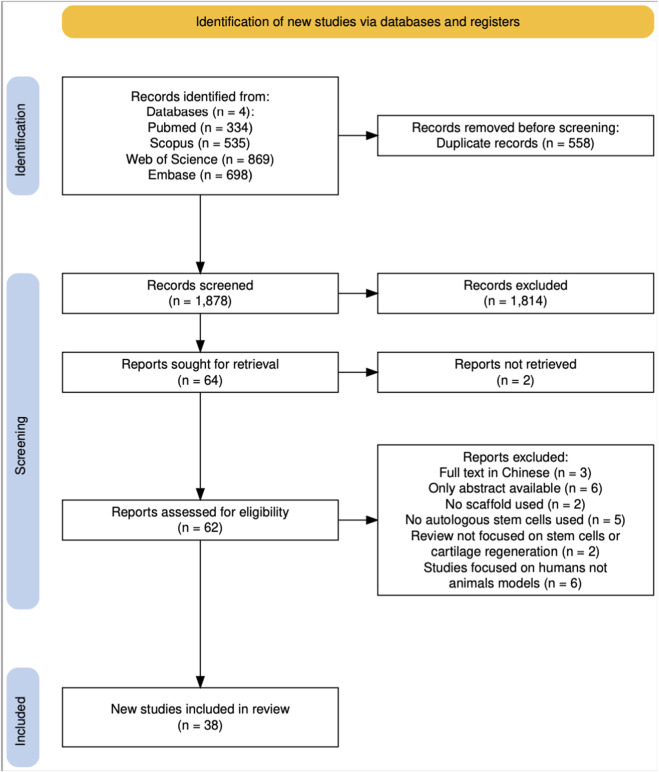
Flow-chart illustrating screening and selection of the articles (Had et al., 2022).

The initial search across four databases yielded a total of 2,436 records. After removing duplicates, 1,878 unique articles remained. Title and abstract screening performed using the Rayyan platform identified 64 potentially relevant studies. Following full-text assessment, 62 articles were retained for comprehensive evaluation, of which 38 met the inclusion criteria for the final analysis. The screening and selection process is summarized in Figure 2, presented as a PRISMA-ScR (Preferred Reporting Items for Systematic Reviews and Meta-Analyses extension for Scoping Reviews) flow diagram (Had et al., 2022). The collected data were synthesized and presented in both tabular and narrative form.

## 3 Preclinical results

Table 1 was compiled from the extracted data from 38 articles, and the results of its analysis are presented in the following sections.

**TABLE 1:** Table presenting the extracted data, categorized by the type of scaffold used.

Scaffold	Source	Concentration/Passage used	Methods of analysis	Observations	Authors
collagen I gel/scaffold	BM-MSCs and periosteal-derived mesenchymal cells (POC)/tibia	not reported	After 2, 4, 12, 24 weeks: Staining technique: Toluidine blue	The scores of the 2 cell types were similar, except for that for surface regularity. The surface of the cartilage that was formed by the POC became more irregular compared with that by BMMC.	Wakitani and Yamamoto (2002)
	Cartilage-derived morphogenetic protein 1 (CDMP1)-transfected BM-MSCs/tibia	1x10^6^ cells/passage 3	After 2, 4, 8 weeks: cell morphology of reconstitution of subchondral bones; Staining technique: Safranin O, Fast Green; surface regularity; Immunohistochemistry (IHC) (CDMP1)	After immunostaining, approximately 20% of the cells reproducibly expressed the transgene. CDMP1-transfected BMMCs showed enhanced expression of aggrecan and Col2a1 with decreased expression of Col1a2 during cell culturing	Katayama et al. (2004)
	BM- MSCs/iliac crest	4x10^5^ cells mm^-1^/1 passage	After 6 months: RT-PCR; Quantun Dots labelling with Qtracker cell labeling kit 605; Staining technique: Toluidine blue; Sulfated glycosaminoglycans (GAGs) content quantification using 1,9-dimethyl-methylene blue dye; tissue sample Apoptosis (TUNEL Assay)	About proteoglycan content, cell count, gel contraction, apoptosis, compressive properties, and progress of chondrogenic differentiation, a differentiation period of 14 days *in vitro* was considered optimal. After 6 months *in vivo*, the defects treated with preMSC-gels showed significantly better histologic scores with morphologic characteristics of hyaline cartilage	Zscharnack et al. (2010)
	BM-MSCs/tibia	not reported/passage 1	After 1, 2, 4, 8 weeks: bromodeoxyuridine (BrdU)-positive cells enumeration and histological grading; RT-PCR	Enumeration of BrdU-positive cells showed a significant increase in the BMP0-1 group compared with the other groups. Similarly, histologic scores in the BMP0-1 group were superior for up to 8 weeks. Finally, RT-PCR findings revealed that immediate BMP-2 administration enhanced chondrogenic differentiation	Mimura et al. (2011)
	BM-MSCs/tibia	1 × 10^6^ cells mL^-1^/passage 1	After 6, 12, 24 weeks: Staining technique: Toluidine blue; Safranin-O and IHC (collagen I/II); histomorphometrically analysis of transplanted cells	Quantitative histological evaluation showed a statistically significantly higher degree of new cartilage area in the MSC groups at 12 and 24 weeks postoperatively compared to the control group	Araki et al. (2015)
collagen I/III membrane	BM-MSCs/tibia	1.7 × 10^6^ cells/3 passage	After 8 weeks: examined for surface irregularities, color, and the amount of defect filling; Staining technique: Safranin O; Fast green; IHC (collagen II)	All membranes were found to cover the defect area 8 weeks postoperatively. Histomorphological scoring revealed significantly higher values in MSC-treated defects when compared to membrane treatment or empty defects. Histomorphometric analysis showed larger GAG/collagen type II-positive areas in the MSC-treated group compared to other groups	Jung et al. (2009)
	Adipose derived MSCs/hip fat BM-MSCs/iliac crest	1.7 × 10^6^ cells/not reported	After 6 months: MRI	The treatment groups (ASC, BMSC, unseeded scaffold) showed MRI profiles closer to reference cartilage	Radke et al. (2023)
collagen I/II gel/scaffold	BM-MSCs/iliac spine	2 × 10^6^ cells L^-1^/3 passage	After 12, 24 weeks: Staining technique: H&E; Alcian blue; Safranin O; IHC (collagen I and II)	The defect space in the experimental group was filled with new cartilage tissues, finely integrated into surrounding normal cartilage. The lamellar scaffold of ß-TCP/col I/col II was gradually degraded and absorbed, while new cartilage tissue formed	Lv and Yu (2015)
	BM-MSCs/femurs, humerus	1 × 10^6^, 5x10^5^ cells mL^-1^/3 passage	DNA Quantification using Hoechst dye; GAG Quantification by dimethyl methylene blue; AP Activity using p-nitrophenylphosphate substrate solution; qRT-PCR for collagen type I, II, and X, aggrecan, Sox9, and glyceraldehyde-3-phosphate dehydrogenase	1) Cells in collagen blend hydrogels are viable and contract gels over time 2) Collagen blend hydrogels increased GAG production 3) Collagen blend hydrogels did not promote an increase in AP activity 4) Sox9 gene expression was upregulated in the Col I/II hydrogels compared to Col I gels 5) Histological staining revealed that the repair tissue from Col I/II hydrogels with autologous MSCs matched surrounding articular cartilage 6) Col I/II hydrogels repaired cartilage defects significantly better than Col I hydrogels or empty defects	Kilmer et al. (2020)
collagen II scaffold	BM-MSCs/humerus	2x10^6^ cells per 100 μL/not reported	After 8, 24 weeks: Staining technique: H&E; Alcian blue; IHC (collagen II)	After 2 months, chondrocyte-like cells with lacuna structure and corresponding ECM were found in the repaired sites without apparent inflammation. After 24 weeks, we could easily find cartilage structure the same with normal cartilage in the repair site	Chen et al. (2011)
	BM-MSCs/iliac bone	5x10^6^ per scaffold/3 passage	After 12 and 24 weeks: Staining technique: H&E; Safranin O and IHC (collagen II); Morphological macroscopic evaluation; MRI	The defects in the blank control and non-CCZ groups were filled with fibrous tissue, while the cartilage layer of the CCZ group was mainly repaired by hyaline cartilage at 24 weeks postoperatively. The superior repair outcome of the CCZ group was confirmed by MOCART and O’Driscoll score	Huang et al. (2021)
three-dimensional poly-lactic-glycolic (PLGA) acid	BM-MSCs/head of humerus	5x10^6^ or 1x10^7^ cells cm^-3^/not reported	After 4, 12 weeks: macroscopically; Staining technique: Toluidine blue; Safranin-O	The structure of the novel PLGA scaffolds provided architectural support for the differentiation of progenitor cells and demonstrated successful induction of *in vivo* chondrogenesis	Uematsu et al. (2005)
​	BM-MSCs/iliac crest	1x10^6^ cells scaffold^-1^/3 passage	After 12 weeks: Staining technique: H&; Toluidine blue; Safranin O and IHC (collagen I/II); RT-PCR for collagen I/II and aggrecan	All scaffolds with cells exhibited better effects compared with the blank group; PLGA porous scaffolds have positive efficacy to help osteochondral healing, and the addition of external BMSCs is helpful for cartilage repairing	Pan et al. (2015)
	BM-MSCs/sternum	5x10^6^ cells/not reported	After 6, 12 months: Staining technique H&E; Safranin O and IHC (collagen II)	Evidence of regeneration of hyaline quality tissue was observed at 6- and 12-month post-treatment. Cartilage and BM-derived mesenchymal stromal cells (MSC), but not those derived from fat, resulted in the best quality of new cartilage	Caminal et al. (2016)
​	BM-MSCs/tibia	1x10^6^ cells cm^-2^/3 passage	After 6, 12, 24 weeks: Staining technique: Safranin O; Fast green	The comparison of two categories, surface zone repair and deeper zone filling/remodeling, indicated that MSCs/PLGA-GCH implantation could enhance cartilage repair, especially in the surface layer (hyaline cartilage zone)	Fan et al. (2006)
	BM-MSCs/iliac crest	10^5^ cells/scaffold/2 passage	After 6, 12 weeks: H&E, Staining technique: Alcian blue; Safranin O and IHC (collagen II); Sulphated Glycosaminoglycan (sGAG) Production Assay; RT-PCR - primers: collagen I/II/IX, aggrecan, sox-9	Significantly higher cell proliferation in PLGA/fibrin construct was noted.PLGA/fibrin construct exhibited better gene expression in all profiles and showed significantly higher relative sGAG content at each time point	Abdul Rahman et al. (2015)
	BM- MSCs/not reported	not reported	After 12 weeks: Staining technique: Toluidine blue, H&E, safranine-O, Masson’s and Goldner’s trichrome, immunolabeling with antibodies for collagen I, collagen II, aggrecan	No improvement of the tissue quantity or quality could be achieved by increasing the cell load of the implant with cells fixed by fibrin glue	Wege et al. (2010)
hyaluronic acid (HA)	BM-MSCs/Ilium	5x10^3^ cells cm^-2^/passage 3	After 4, 12 weeks: Staining technique: H&E; Toluidine blue; Safranin O and IHC (collagen II; CD44)	The experimental group at 12 weeks after surgery showed well-repaired cartilage tissue both microscopically and histologically, resembling the articular cartilage of the surrounding structure	Kayakabe et al. (2006)
	BM-MSCs/iliac crest	5x10^7^ cells mL^-1^/not reported	After 4,8, 12 weeks: gross appearance of the defects; slides graded semi-quantitatively, using the modified ICRS scoring system based on toluidine blue staining	In sECM-only group, hyaline cartilage with zonal architecture filled the defect at 12 weeks, but an interface between repaired and adjacent host cartilage was evident. In the MSCs and sECM group, defects were completely filled with elastic, firm, translucent cartilage at 12 weeks and showed superior integration of the repair tissue with the normal cartilage	Liu et al. (2006)
	BM-MSCs, MSCs/iliac crest	6x10^6^cells mL^-1^)/2 passage	After 12, 24 weeks: H&E, Staining technique: Toluidine blue; H&E; IHC for collagens I, II, and X, MMP-13, and IL-1b	BMC-HA treatment showed a greater repair ability in inhibiting OA progression compared to MSCHA, leading to a reduction of inflammation in cartilage, meniscus, and synovium	Desando et al. (2016)
	BM-MSCs/iliac crest	1x10^7^ cells cm^-2^/3 passage	After 48 weeks: Staining technique: H&E	High degree of filling was obtained, but there was no statistically significant difference between the two treatments. No hypertrophy was observed by either method	Løken et al. (2008)
polylactic acid (PLA) scaffold	BM-MSCs/not reported	1. x10^7^ cells in 0.3 mL/passage 2	After 3, 6 months: Staining technique: GFP (labeling of BM-MSCs) and IHC (collagen II); RT-PCR for procollagen II and aggrecan	In experiment, 11 of 16 defects were completely repaired by hyaline cartilage and cancellous bone, whereas in Ctrl 1, 11 were repaired by fibrocartilage and cancellous bone. No obvious repair was observed in Ctrl 2 and Ctrl 3	Zhou et al. (2006)
	BM-MSCs/tibia, femur	1 million cells in 500 uL/not reported	After 1, 4, 12, 24 weeks: gross morphology; Staining technique: H&E; Safranin O; Fast green and IHC (collagen II); PCR for cell viability	The PLA matrix was still present in the defects at 24 weeks after transplantation of the construct. During the time passage, transplanted MCs numbers decreased from 7.8105 at 1 week, to 3.5105 at 4 weeks, and to 3.8104 at 12 weeks. Transplanted MCs were not detectable at 24 weeks	Oshima et al. (2009)
Collagen Poly-Vinyl-Alcohol (PVA) scaffold	BM-MSCs/femur	400,000 cells cm^2^/not reported	After 12 weeks: morphologic features; Staining technique: IHC (collagen II)	Histology observation showed that the MSCs/collagen-PVA repair group had better chondrocyte morphology, continuous subchondral bone, and much thicker newly formed cartilage compared with the control group	Abedi et al. (2011)
	BM- MSCs/not reported	3x10^5^ cells/passage 3	After 7, 14, 21 days: Staining technique: Alcian blue; DAPI and IHC (collagen II); PAS staining	PVA/PCL scaffolds supported the proliferation and chondrogenic differentiation of MSC *in vitro*. The animals treated with cell-seeded PVA/PCL scaffolds showed improved healing of defects compared with untreated control and those which received cell-free scaffolds	Shafiee et al. (2011)
autologous bone marrow mesenchymal stem cell-derived ECM (aBMSC-dECM) scaffold	Chondrocytes/femoral condyle	3 × 10^6^cells mL^-1^/3 passage	After 1, 2, 4 weeks: Live/Dead cell staining kit; Staining technique: Safranin O and IHC (collagen II); RT-PCR; Western blotting analysis of collagen II	After 4 weeks *in vitro* culture, the engineered cartilage in the aBMSC-dECM scaffold group formed thicker cartilage tissue with more homogeneous structure and higher expressions of cartilaginous gene and protein compared with the atelocollagen scaffold group. Furthermore, the engineered cartilage based on the aBMSC-dECM scaffold showed better cartilage formation in terms of volume and homogeneity, cartilage matrix content, and compressive modulus after 3 weeks *in vivo* implantation	Tang et al. (2013)
	BM-MSCs/illiac crest	not reported	After 6, 12 weeks: Staining technique: Safranin O; Masson’s Trichrome; Sirius Red staining; Quit-iT dsDNA kit; dimethylmethylene blue (DMMB) colorimetric assay analysis of GAGs	Better repair of cartilage defects was observed in the aBMSC-dECM scaffold group than in the BMS group. The glycosaminoglycan and DNA content, the distribution of proteoglycan, and the distribution and arrangement of type II and I collagen fibers in the repaired tissue in the aBMSC-dECM scaffold group at 12 weeks after surgery were similar to that surrounding normal hyaline cartilage	Tang et al. (2014)
	BM-MSCs/iliac crest	not reported	After 6 months: minipigs: MRI; Staining technique: H&E; Safranin O; Masson’s trichrome; gross observation; GAG content using the dimethyl methylene blue colorimetric assay; DNA content by the Quit-iT dsDNA kit; After 6, 72, 238 h: rabbits: gross observation; CFU-F assay	In the rabbit model, the macroscopic appearance of the exudate of the healing wounds in the ECM group showed less fibrosis, and the histology showed more evenly distributed chondrocytes compared with the BMS group. The CFU-F assay showed that the number of bone MSCs in the ECM group was approximately was twice that of the BMS group. In the minipig model, the macroscopic appearance and magnetic resonance imaging (MRI) findings of the ECM group were improved when compared with the BMS group	Tang et al. (2019)
	BM-MSCs/not reported	not reported/3 passage	After 6,12 weeks: Staining technique: Safranin O; IGC (collagen1/22/X; VEGF); Microscopic evaluation	ECM-hydrogel group showed improved fill/integration and more cartilage-like matrix features vs. agarose + chondrocytes and microfracture controls by 12 weeks	Wei et al. (2024)
oligo-poly-ethylene glycol-fumarate (OPF) scaffold	BM-MSCs/tibia	10 million cells ml^-1^/not reported	After 12 weeks: Staining technique: H&E; Safranin O; Fast green; Masson’s trichrome	With the implantation of scaffolds alone, newly formed chondral tissue had an appearance of hyaline cartilage with zonal organization and intense staining for glycosaminoglycans. The addition of MSCs, especially with TGF-beta1-loaded GMPs, facilitated subchondral bone formation	Guo et al. (2010)
Gelfoam (Gelatine sponge)	BM-MSCs/iliac crest	2x10^6^ cells cm^-2^/passage 3	After 4, 8, 12 weeks: radiographs; Staining technique: H&E; Masson’s trichrome	At 12 weeks, 3/5 femurs in experimental group were united, but 1/5 in control group was united. The expression of BMP-2 was significantly higher at 4, 8 weeks, the expressions of BMP-4 and BMP-7 were significantly higher at 8 and 12 weeks, and the expression of VEGF and RANKL were significantly higher at all time points in experimental group	Lee et al. (2011)
Polycaprolactone-Hydroxyapatite (PCL-HA) scaffold	BM-MSCs/iliac spine	not reported	After 12 weeks: Staining technique: H&E; Safranin O and IHC (collagen I/II)	Experimental group showed excellent vertical and lateral integration with host bone, but incomplete cartilage regeneration and matrix accumulation	Wei et al. (2015)
silk-nanoCaP scaffolds	BM-MSCs/femur	5x10^6^ cells ml^-1^/2 passage	After 4 weeks: Staining technique: H&E; Masson’s trichrome; Safranin O and IHC (collagen II)	Histological and immunohistochemical analysis showed that collagen II positive cartilage and glycosaminoglycan regeneration presented in the silk layer, and *de novo* bone ingrowths and vessel formation were observed in the silk-nanoCaP layer	Yan et al. (2015)
Fibrin scaffold	BM-MSCs/femur	1x10^7^, 2x10^7^ cells mL^-1^	After 8 weeks: Staining technique: H&E; Safranin O and IHC (collagen I/II; aggrecan)	In the experimental groups, Group B (fibrin and cells) had the highest average morphological score, and the highest percentage filling of the defect area compared to all other groups. There is evidence that dense rBMSC aggregates (Group B) may be beneficial for osteochondral tissue engineering. The cells were regular cartilage-like cells and did not exhibit any hyper-cellularity. Since there was no strong collagen I stain, it may be indicative of a hyaline-like cartilage	Sridharan et al. (2017)
Fibrin glue hydrogel enclosed by electrospun nanofibrous mats	BM-MSCs/iliac crest	2x10^7^ cells mL^-1^/5 passage	After 24 weeks: Staining technique: H&E; Safranin O; Fast Green; immunofluorescence (COL1A1, COL2A1, COL10A1); MRI	Cellular implant groups improved repair vs. microfracture and acellular controls. BMSC-derived implants showes more hyaline-like phenotype (COL2A1-high with minimal COL1A1/COL10A1); BMSC groups stiffness lower than intact cartilage	Lee et al. (2025)
PVA/CS composite hydrogel	BM-MSCs/femur	50,000 cells/2 passage	After 12 weeks: Staining technique: H&E; Safranin O and IHC (collagen II); Macroscopic cartilage evaluation	The Hydrogel with PVA/CS ratio of 6:4 exhibited the best mechanical properties; it also showed stable physical and chemical properties with porosity and over 90% water content. Furthermore, it demonstrated no cytotoxicity and was able to promote cell proliferation	Peng et al. (2019)
multi-layered chitosan-gelatin (CG) scaffold	BM-MSCs/not reported	1 × 10^6^ cells mL^-1^/3 passage	After 4 months: Staining technique: Safranin O and IHC (collagen II/X); Morphological, macroscopic evaluation	*In vivo* cartilage formation occurred in both multi-layered and randomly aligned scaffolds treated with and without cells and was shown to be of hyaline phenotype on immunostaining. The defects treated with multi-layered + cells, however, showed significantly thicker cartilage formation than the randomly aligned scaffold	Rajagopal et al. (2020)
Polyglycolic acid-hyaluronan (PGA-HA) scaffold	BM-MSCs/femur	2.5 × 10^6^ cells/2 passage	After 90 days: Staining technique: Verhoeff’s hematoxylin/green trichrome; PAS; alcian blue; Macroscopic evaluation	Untreated defects filled more macroscopically but were more fibrous. The scaffold groups had more hyaline cartilage. MSC group showed undifferentiated connective tissue at defect edges and no detectable systemic inflammatory activation	Berounský et al.(2023)

### 3.1 Different scaffold types

The analyzed studies suggest that scaffolds significantly improve cell transplantation outcomes in chondral defects, by providing biomechanical strength, creating a suitable microenvironment for cellular synthesis, and facilitating the transport and absorption of growth factors from surrounding tissues (Mimura et al., 2011).

Across the analyzed studies, many different, both natural and synthetic, scaffolds were identified. Collagen/ECM scaffolds were the most commonly used and present in various forms such as collagen I gel (Araki et al., 2015; Mimura et al., 2011; Abdul Rahman et al., 2015; Filardo et al., 2013; Katayama et al., 2004; Matsumoto et al., 2010; Wakitani and Yamamoto, 2002; Yamasaki et al., 2014; Zscharnack et al., 2010), collagen I/III membrane (Jung et al., 2009; Radke et al., 2023), collagen I/II gel (Kilmer et al., 2020; Lv and Yu, 2015), collagen II scaffold (Chen et al., 2011; Huang et al., 2021), as well as collagen scaffolds with addition of polycaprolactone (PCL) (Shafiee et al., 2011), Poly-Vinyl-Alcohol (PVA) (Shafiee et al., 2011; Abedi et al., 2011; Peng et al., 2019), autologous bone marrow mesenchymal stem cell-derived extracellular matrix (aBMSC-dECM) (Tang et al., 2014; Tang et al., 2019; Tang et al., 2013; Wei et al., 2024) or beta-tricalcium phosphate (β-TCP) (Lv and Yu, 2015).

As collagen scaffolds are composed of natural ECM with regular alignment of collagen fibers, they mimic the native cartilage environment and provide suitable conditions for MSCs to differentiate into cartilage producing chondrocytes while at the same time ensuring good cell retention by maintaining the cells in place (Araki et al., 2015). The presence of substrate-bound collagen within the scaffold not only enhances the recruitment of MSCs via chemotaxis but also directs them towards the central region of the defect, as cells migrate along a concentration gradient (Mimura et al., 2011). Usage of aBMSC-dECM minimizes the risk of host immune response and inflammation. The quick and complete degradation of aBMSC-dECM enhances the transportation of nutrients and metabolites to the defect site (Tang et al., 2014; Tang et al., 2019; Tang et al., 2013). On the other hand, a significant challenge is their failure to bond with adjacent cartilage, which can lead to surface irregularities and increased susceptibility to physiological pressure (Abedi et al., 2011). Because of this, Wei et al. processed a BMSC-derived ECM scaffold into an injectable hydrogel, enabling it to conform to irregular defect surfaces (Wei et al., 2024). Also, pure collagen scaffolds often lack mechanical strength and are susceptible to collapse or deformation of their 3D structure under load. Thus, composite materials are added to strengthen the scaffolds. However, simultaneously heterogeneity is introduced, where biological and mechanical properties may vary across locations.

Another class of scaffolds employed consisted of synthetic biodegradable polyesters, including polylactic acid (PLA) (Oshima et al., 2009; Zhou et al., 2006), poly-lactic-glycolic acid (PLGA) (Abdul Rahman et al., 2015; Caminal et al., 2016; Fan et al., 2006; Pan et al., 2015; Uematsu et al., 2005; Wege et al., 2010), polycaprolactone-hydroxyapatite (PCL-HA) (Abdul Rahman et al., 2015), and polyglycolic acid-hyaluronan (PGA-HA) scaffolds (Berounský et al., 2023).

These scaffolds may be engineered to provide high mechanical strength and shape stability which is crucial for defect geometry during the healing process. Also, their structure may be precisely altered to fulfill the researchers needs such as pore sizes to provide optimal cell infiltration and nutrient diffusion, shape to mimic the natural orientation, and layer specific composition to provide controlled heterogeneity in specific layers. Their structure prevents leakage of the cells into the defected site. The presence of pores in microsponge-like scaffold enhances the efficiency of cell seeding and retention (Uematsu et al., 2005). Practically, several groups improve intraoperative cell retention by combining scaffolds with autologous plasma/fibrinogen-based hydrogels, which can polymerize *in situ* and help immobilize cells within porous matrices (Berounský et al., 2023). However, the degradation of such scaffolds needs to be controlled as common polymers tend to degrade into acidic byproducts which may lower the local pH. Also, those scaffolds lack materials that mimic the natural ECM and might induce the cells differentiation into fibrocartilage formation which in this area would be inferior to hyaline cartilage (Fan et al., 2006).

Additionally, natural materials such as hyaluronic acid (HA) gel (Desando et al., 2016; Kayakabe et al., 2006; Liu et al., 2006; Løken et al., 2008), gel foam (gelatine sponge) (Lee et al., 2011), fibrin (Radke et al., 2023; Sridharan et al., 2017; Lee et al., 2025) and multilayered chitosan-gelatin (CG) (Rajagopal et al., 2020) scaffolds were examined.

These scaffolds promote cell viability, enhance matrix diffusion, and ensure convenient intraoperative manipulation. Also, HA gel is considered to enhance MSCs differentiation into chondrocytes and migration rate enhancement due to their lubricating and anti-inflammatory properties (Kayakabe et al., 2006). However, their limited mechanical strength under load confines their role mainly to that of carriers rather than load-bearing scaffolds.

Across studies, scaffold chemistry appears to shape the quality of the repair matrix and the balance between chondrogenic maturation and subchondral bone response. Natural and ECM-rich collagen-based scaffolds consistently promoted intense proteoglycan deposition and the formation of a collagen II-positive matrix, supporting more hyaline-like cartilage at mid-term endpoints (Kilmer et al., 2020; Chen et al., 2011). Composite scaffolds that integrate ECM with structural reinforcement (e.g., collagen-based scaffolds incorporating mineral phases or zonal architectures) were generally effective in achieving defect fill and integration while maintaining construct stability during remodeling (Lv and Yu, 2015; Huang et al., 2021). In contrast, mechanically robust synthetic scaffolds often ensured excellent defect stabilization and osseous integration but, in some models, exhibited incomplete cartilage matrix accumulation at comparable time points (Lee et al., 2011). A useful mechanistic interpretation across biomaterial classes is that outcomes are not driven solely by “bioactivity” of the material, but also by how well the implant conforms to defect geometry and stabilizes the tissue interface. For example, injectable ECM-based hydrogels represent one strategy to reduce interfacial gaps and micromotion - in a rabbit model an autologous BMSC-derived ECM hydrogel demonstrated improved integration and cartilage-like matrix features compared with agarose and microfracture controls (Wei et al., 2024).

These observations suggest that bioactive, ECM-mimetic cues are critical for proper chondrogenic matrix organization, whereas purely synthetic or highly osteoconductive scaffolds may require additional biological augmentation to achieve hyaline-like cartilage.

From a clinical perspective, it appears that the mechanical durability and integration of the implant with the surrounding tissues are more critical than complete biological remodeling toward native cartilage. As long as the implant remains stable and biomechanically comparable to the adjacent tissue, full biological transformation may not be strictly necessary.

A comparative summary of the scaffold categories used across the included studies is provided in Table 2.

**TABLE 2:** Comparison of scaffold categories (natural vs. synthetic, including composites) used for MSC-based cartilage repair in the included preclinical studies.

Scaffold category	Representative materials used in included studies	Key advantages	Key limitations	Frequency of use across included studies (n = 38)	Refrences
Natural	Collagen-based scaffolds (collagen I gels/scaffolds; collagen I/II gels; collagen I/III membranes; collagen II scaffolds), fibrin, gelatin sponge (Gelfoam), hyaluronic acid (HA), chitosan-gelatin (CG), autologous BMSC-derived ECM (aBMSC-dECM)	High biocompatibility and bioactivity; supports MSC adhesion and chondrogenic differentiation; biomimetic microenvironment; generally favorable integration with native tissue	Lower/variable mechanical strength; batch-to-batch variability and less predictable degradation; limited ability to independently tune mechanics vs. bioactivity; potential immunogenicity depending on source/purification	22/38 studies	Araki et al. (2015), Mimura et al. (2011), Katayama et al. (2004), Wakitani and Yamamoto (2002), Zscharnack et al. (2010), Jung et al. (2009), Radke et al. (2023), Kilmer et al. (2020), Lv and Yu (2015), Chen et al. (2011), Huang et al. (2021), Tang et al. (2014), Tang et al. (2019), Tang et al. (2013), Wei et al. (2024), Desando et al. (2016), Kayakabe et al. (2006), Liu et al. (2006), Løken et al. (2008), Lee et al. (2011), Sridharan et al. (2017), Rajagopal et al. (2020)
Synthetic	Poly (lactic-co-glycolic acid) (PLGA), polylactic acid (PLA), oligo (poly (ethylene glycol) fumarate) (OPF), poly (vinyl alcohol) (PVA)	Reproducible manufacturing; tunable porosity, mechanics and degradation; good structural support; scalable and easier quality control	Limited intrinsic cell-adhesive cues (often requires functionalization or additives); polymer degradation by-products (e.g., acidic PLGA/PLA) may affect local pH; bioactivity may be insufficient without ECM-mimetic modification	7/38 studies	Oshima et al. (2009), Zhou et al. (2006), Caminal et al. (2016), Pan et al. (2015), Uematsu et al. (2005), Wege et al. (2010), Guo et al. (2010)
Composite/Hybrid	Collagen-PVA, PVA/chitosan (PVA/CS) composite hydrogel, polycaprolactone-hydroxyapatite (PCL-HA), silk-nanoCaP	Combines mechanical support of synthetic/inorganic phases with bioactivity of natural components; can improve stiffness, handling and durability while maintaining a pro-chondrogenic environment	More complex fabrication and characterization; potential interfacial issues/phase separation; harder to standardize; degradation kinetics and by-products may be more difficult to predict	9/38 studies	Abdul Rahman et al. (2015), Shafiee et al. (2011), Abedi et al. (2011), Peng et al. (2019), Fan et al. (2006), Berounský et al. (2023), Lee et al. (2025), Wei et al. (2015), Yan et al. (2015)

### 3.2 MSCs sources

MSCs sources were diverse across studies listed in Table 1, with rabbit MSCs being the most frequent (Abdul Rahman et al., 2015; Filardo et al., 2013; Katayama et al., 2004; Wakitani and Yamamoto, 2002; Yamasaki et al., 2014; Kilmer et al., 2020; Chen et al., 2011; Shafiee et al., 2011; Abedi et al., 2011; Peng et al., 2019; Tang et al., 2014; Tang et al., 2019; Tang et al., 2013; Wei et al., 2024; Oshima et al., 2009; Fan et al., 2006; Pan et al., 2015; Uematsu et al., 2005; Kayakabe et al., 2006; Liu et al., 2006; Løken et al., 2008; Lee et al., 2011; Jia et al., 2015; Wei et al., 2015; Yan et al., 2015) followed by MSCs extracted from mini pigs (Jung et al., 2009; Radke et al., 2023; Huang et al., 2021; Tang et al., 2019; Berounský et al., 2023; Lee et al., 2025), sheep (Zscharnack et al., 2010; Wege et al., 2010; Desando et al., 2016), rats (Filardo et al., 2013; Sridharan et al., 2017), pigs (Zhou et al., 2006), horses (Filardo et al., 2013), monkeys (Araki et al., 2015), dogs (Lv and Yu, 2015), and mice (Filardo et al., 2013).

This distribution suggests that rabbits dominate the preclinical experimental models for studying cartilage repair. This may be due to their cartilage dimensions: rabbits have larger joints than smaller laboratory animals, but smaller than those of large animal models, which makes surgical procedures in joints easier and facilitates postoperative care (Moran et al., 2016). However, due to their thinner cartilage, treatment effectiveness may be overestimated comparing to human-scale joints, highlighting the need for a shift towards larger-animal models (Yamasaki et al., 2014).

### 3.3 Heterogeneity in cell seeding and passage

The scaffold seeding, which is placing living cells onto or into a scaffold, was described using three quantification methods. The first one being cells/cm3 with values ranging from 5 x 10^5^ to 5 x 10^7^, reflecting substantial variability across studies (Zscharnack et al., 2010; Kilmer et al., 2020; Zhou et al., 2006; Fan et al., 2006; Berounský et al., 2023; Desando et al., 2016; Liu et al., 2006; Sridharan et al., 2017; Lee et al., 2025; Yan et al., 2015; Guo et al., 2010). The second quantification method implemented cells/cm2 units with values ranging from 5 x 10^3^ to 4 x 10^5^ (Abedi et al., 2011; Tang et al., 2013; Kayakabe et al., 2006). Lastly, the total number of cells per implant was used which varied from an order of magnitude of 2 × 10^3^ to 5 x 10^7^ (Araki et al., 2015; Abdul Rahman et al., 2015; Katayama et al., 2004; Matsumoto et al., 2010; Yamasaki et al., 2014; Jung et al., 2009; Radke et al., 2023; Lv and Yu, 2015; Chen et al., 2011; Huang et al., 2021; Shafiee et al., 2011; Peng et al., 2019; Oshima et al., 2009; Caminal et al., 2016; Pan et al., 2015; Løken et al., 2008; Lee et al., 2011; Jia et al., 2015). Several animal studies did not report either seeding density or total implanted cell number (Mimura et al., 2011; Filardo et al., 2013; Wakitani and Yamamoto, 2002; Tang et al., 2014; Tang et al., 2019; Wege et al., 2010).

Across animal studies listed in Table 1, no consistent relationship was observed between higher seeding density or higher total number of cells and superior chondrogenic outcomes. These findings do not necessarily reflect the ineffectiveness of the treatment but rather point out that the results may be confounded by other factors such as scaffold volume and porosity, species and defect size, follow-up time, and implantation site. Interestingly, Berounský et al. showed that delivery of approximately 2.5 × 10^6^ autologous early-passage (P2) BM-MSCs in a PGA-HA scaffold produced more hyaline-like repair tissue histologically, even though macroscopic defect fill was not superior to untreated controls at 90 days (Berounský et al., 2023). Nevertheless, one study highlighted dose-dependent effects. Higher-density delivery and/or dense MSC aggregates within fibrin-based carriers was associated with improved defect fill and superior morphological and histological outcomes *in vivo* (Yan et al., 2015). Overall, the evidence supports a context-dependent relationship, in which cell loading enhance repair in permissive carriers (e.g., hydrogels) but does not guarantee improved outcomes if scaffold permeability, defect size, or the local microenvironment are not optimized.

For future cross-study comparability, studies should normalize the number of cells per defect volume (for example, cells/mm^3^) and report the scaffold porosity.

Additionally, 20 studies did not specify the cell passage number (Filardo et al., 2013; Matsumoto et al., 2010; Wakitani and Yamamoto, 2002; Yamasaki et al., 2014; Radke et al., 2023; Kilmer et al., 2020; Lv and Yu, 2015; Chen et al., 2011; Abedi et al., 2011; Tang et al., 2014; Tang et al., 2019; Oshima et al., 2009; Zhou et al., 2006; Caminal et al., 2016; Uematsu et al., 2005; Wege et al., 2010; Liu et al., 2006; Løken et al., 2008; Jia et al., 2015; Wei et al., 2015; Guo et al., 2010), which is the count of how many times a cell culture has been sub-cultured to prevent overgrowth. Most investigations implemented cells at early passages, ranging from passage 0 (P0) (Sridharan et al., 2017) to cells passaged up to 5 times (P5) (Lee et al., 2025; Veronesi et al., 2013). However, most studies conducted 3 cell passages (Abdul Rahman et al., 2015; Jung et al., 2009; Huang et al., 2021; Tang et al., 2013; Wei et al., 2024; Fan et al., 2006; Pan et al., 2015; Kayakabe et al., 2006; Lee et al., 2011; Veronesi et al., 2013), followed by studies with 2 passages (Abdul Rahman et al., 2015; Kilmer et al., 2020; Shafiee et al., 2011; Peng et al., 2019; Berounský et al., 2023; Yan et al., 2015), and 1 passage (Araki et al., 2015; Mimura et al., 2011; Abdul Rahman et al., 2015; Zscharnack et al., 2010).

Passage number was incompletely reported across studies, limiting inference about its contribution to outcomes. Where described, early passage MSCs (commonly P1-P3) predominated and were associated with favorable histological outcomes in multiple constructs, including enhanced collagen II- and GAG-rich repair regions (Jung et al., 2009; Pan et al., 2015).

### 3.4 Cell harvest sources and cell types used

**FIGURE 3:**
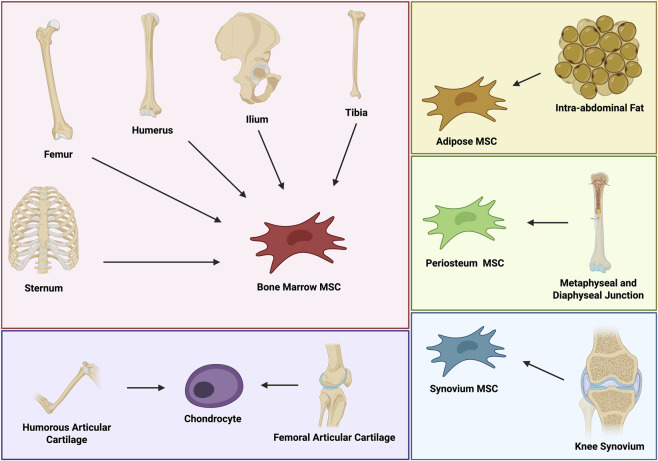
MSCs sources for cartilage repair. This diagram illustrates various sources of MSCs (bone marrow, adipose, synovium, periosteum) and chondrocytes, highlighting their relevance to different anatomical locations (Rytel, 2025a).

Cell harvest sources in articles mentioned in Table 1 varied across the animal models, with bone marrow-derived MSCs (BMSCs) from ilium being the most common (Abdul Rahman et al., 2015; Matsumoto et al., 2010; Yamasaki et al., 2014; Zscharnack et al., 2010; Radke et al., 2023; Lv and Yu, 2015; Huang et al., 2021; Tang et al., 2014; Tang et al., 2019; Wei et al., 2024; Pan et al., 2015; Desando et al., 2016; Kayakabe et al., 2006; Løken et al., 2008; Lee et al., 2011; Lee et al., 2025; Wei et al., 2015), followed by femur (Kilmer et al., 2020; Abedi et al., 2011; Peng et al., 2019; Tang et al., 2013; Oshima et al., 2009; Caminal et al., 2016; Berounský et al., 2023; Liu et al., 2006; Sridharan et al., 2017; Veronesi et al., 2013; Liang et al., 2018), tibia (Katayama et al., 2004; Wakitani and Yamamoto, 2002; Jung et al., 2009; Oshima et al., 2009; Fan et al., 2006; Jia et al., 2015; Guo et al., 2010), humerus (Kilmer et al., 2020; Chen et al., 2011; Abedi et al., 2011; Uematsu et al., 2005), and sternum (Caminal et al., 2016). Chondrocytes were isolated from either femoral articular cartilage (Caminal et al., 2016), humorous articular cartilage (Caminal et al., 2016). Adipose-derived MSCs were extracted from intra-abdominal fat (Filardo et al., 2013) or subcutaneous hip fat (Radke et al., 2023), synovium-derived MSCs from knee synovium (Filardo et al., 2013), and periosteal-derived MSCs from metaphyseal and diaphyseal junction (Filardo et al., 2013), as shown on Figure 3.

The most common cell type used in the studies was BMSCs, followed by chondrocytes (Caminal et al., 2016; Yan et al., 2015), adipose-derived MSCs (Filardo et al., 2013; Caminal et al., 2016), periosteal-derived MSCs (Wakitani and Yamamoto, 2002) and synovium-derived MSCs (Filardo et al., 2013).

The advantage of undifferentiated cells implantation is its capability to have more mitotic divisions in the chondral defect compared to highly differentiated chondrocytes (Wakitani and Yamamoto, 2002). Among the various MSC types, BMSCs are relatively easy to isolate and expand using well-established protocols, making them suitable candidates for *in vivo* experimentation (Pittenger et al., 1999). In contrast, harvesting synovium-derived MSCs requires an additional invasive joint procedure, adding procedural complexity, while the yield of adipose-derived MSCs can vary depending on the donor site, species, and extraction technique (To et al., 2019; Mailey et al., 2014). These factors contribute to reduced predictability and standardization of the results.

An overview of the cell types utilized in the studies and their respective sources of extraction is presented in Figure 3 (Rytel, 2025a). Importantly, all studies mentioned in Table 1 extracted MSCs from the recipient’s own body - therefore, they used them in an autologous transplantation. Allogenic MSC studies were not analyzed in this review due to higher risk of severe adverse effects and less beneficial outcomes after allogenic MSC use (Jeyaraman et al., 2022).

Most studies included in this review employed autologous bone marrow-derived MSCs, limiting direct comparisons between different cell sources. In studies that directly compared autologous sources under similar scaffold and defect conditions, cell origin appeared to influence repair outcomes. In an ovine osteochondral defect model using PLGA scaffolds, cartilage and bone marrow-derived MSCs demonstrated superior cartilages repair of defects compared with adipose-derived cells over a 6–12-month period, suggesting that tissue source may impact long-term hyaline-like cartilage formation (Oshima et al., 2009).

In collagen-based constructs, periosteum-derived and bone marrow-derived cells achieved comparable overall defect repair scores, although surface regularity differed between groups (Wakitani and Yamamoto, 2002). These findings support the conclusion that bone marrow-derived MSCs currently provide the most robust evidence base in this field, whereas alternative autologous sources may require optimized conditioning or scaffold design to achieve comparable durability.

### 3.5 Cell manipulation

In all the papers, MSC cells were culture-expanded prior to implantation. Another technique widely implemented in different experimental settings is the usage of the bone marrow concentrate - bone marrow is harvested from the animal and centrifuged to obtain a concentrate enriched with nucleated cells, including a small fraction of stem cells, which is subsequently implanted into the defect site.

Taking into consideration the analyzed studies, cultured cell use enabled regeneration of larger cartilage defects in terms of area, about twice the size of that observed in the BMSCs concentrate treatment (Veronesi et al., 2013). Apart from that, expansion of MSCs enables precise dosing and scaffold seeding before the implantation. In contrast, BMSC concentrates contain heterogeneous cell populations, making it difficult to determine the extent to which the observed effects can be specifically attributed to MSCs (Wei et al., 2019).

### 3.6 Final duration of the experiment

The duration of the experiments varied across analyzed studies, ranging from 3 weeks (Shafiee et al., 2011) to as long as 12 years (Matsumoto et al., 2010; Yamasaki et al., 2014). Some studies included experiments that lasted 4 weeks (Tang et al., 2013; Yan et al., 2015) and 8 weeks (Mimura et al., 2011; Katayama et al., 2004; Jung et al., 2009; Sridharan et al., 2017). A significant number of experiments was conducted over 12 weeks period (Abdul Rahman et al., 2015; Abedi et al., 2011; Peng et al., 2019; Tang et al., 2014; Wei et al., 2024; Caminal et al., 2016; Fan et al., 2006; Pan et al., 2015; Wege et al., 2010; Berounský et al., 2023; Kayakabe et al., 2006; Liu et al., 2006; Lee et al., 2011; Wei et al., 2015), while others extended to 24 weeks (Araki et al., 2015; Wakitani and Yamamoto, 2002; Lv and Yu, 2015; Chen et al., 2011; Huang et al., 2021; Oshima et al., 2009; Fan et al., 2006; Desando et al., 2016; Løken et al., 2008; Lee et al., 2025; Jia et al., 2015). Other studies included 4 months (Rajagopal et al., 2020) and 6 months (Zscharnack et al., 2010; Radke et al., 2023; Tang et al., 2019; Zhou et al., 2006) experimental periods. The initial repair phase, typically evaluated at around 4 weeks, focuses on cellular activity rather than mature matrix formation. This timepoint is optimal for assessing cell survival, spatial distribution, inflammatory response, early matrix deposition, and the expression of chondrogenic differentiation markers. Histological assessments performed between 8 and 12 weeks provide insight into the progression of extracellular matrix synthesis, organization, and cell activity; however, complete structural and mechanical maturity is generally not yet achieved at this stage. The later stages of healing (typically 16 weeks and 6 months, depending on the animal model), allow assessment of osteochondral remodeling, structural integrity, and long-term cell viability, reflecting tissue stabilization rather than active repair.

Model choice and follow-up duration strongly affect both outcome magnitude and translational interpretability. Many studies evaluate repair at 8–24 weeks, capturing early defect filling and matrix deposition, but this timeframe may not fully reflect long-term tissue stability or ongoing remodeling. Later time points can reveal durability limitations that are not apparent in early assessments. In study by Wakitani et al., the authors demonstrated that collagen-based scaffold autologous MSCs repair strategies exhibited thinning of the repair tissue and reduced metachromasia at later evaluations, indicating that early improvements may not be sustained over time. (Wakitani and Yamamoto, 2002). In a PLA-based scaffold study, the number of implanted cells progressively declined and was reportedly undetectable by 24 weeks, suggesting that long-term outcomes depend primarily on the host tissue remodeling capacity rather than on sustained implant persistence (Oshima et al., 2009). Large-animal studies with extended follow-up (e.g., 6–12 months) are particularly informative, as they better reflect physiological joint loading and cartilage thickness, enabling discrimination between repair strategies that appear comparable in short-term evaluations (Caminal et al., 2016). Without a doubt, standardizing observation periods across studies would improve comparability and understanding of the full healing process.

It should be noted that favorable clinical outcomes, such as absence of swelling and pain, as well as satisfactory function of the operated joint - may not correlate with imaging or histological findings. This suggests that scaffold remodeling toward native cartilage tissue is not the sole factor determining clinical success.

## 4 Preclinical applied research methods

The aim of the following subsection is to outline how the joint tissue was evaluated after implantation of MSCs-scaffold construct. This section describes the methods used to assess cartilage regeneration and the tissue response to treatment, including macroscopic inspection, histological analysis, and any additional examinations performed to determine the quality and integration of the newly formed tissue. Data discussed in this section are also summarized in Table 1.

### 4.1 Histological techniques

Safranin O and Hematoxylin and Eosin (H&E) staining were the most frequently used techniques, reported in 22 (Araki et al., 2015; Abdul Rahman et al., 2015; Jung et al., 2009; Lv and Yu, 2015; Chen et al., 2011; Peng et al., 2019; Tang et al., 2014; Tang et al., 2019; Tang et al., 2013; Oshima et al., 2009; Caminal et al., 2016; Fan et al., 2006; Pan et al., 2015; Wege et al., 2010; Kayakabe et al., 2006; Sridharan et al., 2017; Lee et al., 2025; Rajagopal et al., 2020; Jia et al., 2015; Wei et al., 2015; Liang et al., 2018) and 18 (Abdul Rahman et al., 2015; Jung et al., 2009; Lv and Yu, 2015; Chen et al., 2011; Huang et al., 2021; Peng et al., 2019; Tang et al., 2019; Oshima et al., 2009; Caminal et al., 2016; Pan et al., 2015; Desando et al., 2016; Kayakabe et al., 2006; Lee et al., 2011; Sridharan et al., 2017; Lee et al., 2025; Jia et al., 2015; Wei et al., 2015) analyzed studies, respectively. Toluidine Blue staining was used in 9 studies (Araki et al., 2015; Wakitani and Yamamoto, 2002; Zscharnack et al., 2010; Pan et al., 2015; Uematsu et al., 2005; Wege et al., 2010; Desando et al., 2016; Kayakabe et al., 2006), Masson’s Trichrome staining in 6 (Tang et al., 2014; Tang et al., 2019; Løken et al., 2008; Lee et al., 2011; Liang et al., 2018), Alcian Blue staining in 4 (Abdul Rahman et al., 2015; Lv and Yu, 2015; Chen et al., 2011; Shafiee et al., 2011), and Fast Green staining in 4 studies (Jung et al., 2009; Oshima et al., 2009; Fan et al., 2006; Lee et al., 2025), while Sirius Red staining was applied in 1 study (Tang et al., 2014). Additionally, other techniques such as Periodic Acid-Schiff (PAS) staining (Shafiee et al., 2011), histomorphometric analysis (Araki et al., 2015), and BrdU-positive cell enumeration (Mimura et al., 2011) were also conducted.

The plethora of stains used captures both matrix composition (for example, GAGs) and structure (collagen organization), which aids in the differentiation of hyaline-like cartilage from fibrocartilage. The implementation of histomorphometric analysis and enumeration of BrdU-positive cells provides more quantitative and cell-kinetics context (Mainil-Varlet et al., 2003; Miller and Nowakowski, 1988). However, these techniques were absent in the majority of studies, despite their potential to provide valuable insights into cell division and growth dynamics, which are crucial in MSCs transplantations. Moreover, the applied techniques do not assess potential hypertrophic changes, which may compromise joint integrity and hinder adaptive remodeling (Caplan and Dennis, 2006; De Bari et al., 2001).

### 4.2 Molecular and biochemical techniques

Immunohistochemistry was used to detect CDMP1 (Lv and Yu, 2015), CD44 (Kayakabe et al., 2006), aggrecan (Zhou et al., 2006; Sridharan et al., 2017), collagen X (Rajagopal et al., 2020), collagen I (Lv and Yu, 2015; Tang et al., 2014; Pan et al., 2015; Sridharan et al., 2017; Wei et al., 2015), and most commonly collagen II, which was analyzed in 18 studies (Abdul Rahman et al., 2015; Jung et al., 2009; Lv and Yu, 2015; Huang et al., 2021; Shafiee et al., 2011; Abedi et al., 2011; Peng et al., 2019; Tang et al., 2014; Tang et al., 2013; Oshima et al., 2009; Zhou et al., 2006; Caminal et al., 2016; Pan et al., 2015; Kayakabe et al., 2006; Sridharan et al., 2017; Rajagopal et al., 2020; Wei et al., 2015; Yan et al., 2015). Reverse Transcription Polymerase Chain Reaction (RT-PCR) was performed for collagen I and II in 2 studies (Abdul Rahman et al., 2015; Pan et al., 2015), for aggrecan in 3 studies (Mimura et al., 2011; Abdul Rahman et al., 2015), and for collagen IX (Abdul Rahman et al., 2015) and SOX-9 (Abdul Rahman et al., 2015) in one study each. In addition, glycosaminoglycan (GAG) quantification was carried out in 4 studies (Abdul Rahman et al., 2015; Zscharnack et al., 2010; Kilmer et al., 2020; Tang et al., 2014), DNA quantification in 3 studies (Kilmer et al., 2020; Tang et al., 2014; Tang et al., 2019), apoptosis detection using the TUNEL assay in 2 studies (Abdul Rahman et al., 2015; Zscharnack et al., 2010), and Western blot analysis in 1 study (Tang et al., 2013).

Pairing collagen II with Safranin O staining supports the identification and localization of hyaline matrix (Solchaga et al., 2001). Also, pairing GAG content with DNA amount allows for approximation of the cell number present and the calculation of matrix (GAG) amount produced by each cell (Sanchez-Adams and Athanasiou, 2010). This ratio enables comparison between samples despite possible cell density variations and assessment of the chondrogenic activity of the implanted cells. On the other hand, these techniques require tissue homogenization, which can obscure region-specific differences, for example, superficial versus deep zones (Kock et al., 2012).

### 4.3 Imaging techniques

In terms of imaging techniques used in the analyzed articles, Magnetic Resonance Imaging (MRI) was utilized on large animals in 4 studies (Radke et al., 2023; Huang et al., 2021; Tang et al., 2019; Lee et al., 2025), arthroscopy in 1 (Yamasaki et al., 2014) and radiography also in 1 study (Lee et al., 2011). In one study (Yamasaki et al., 2014) biopsy was collected to enable subsequent detailed analysis.

Despite its high operational cost, MRI provides a non-invasive method for longitudinal monitoring of cartilage repair. Owing to the joint size and anatomy of large animal models, which closely resemble those of humans, this approach possesses strong translational relevance. Moreover, MRI enables repeated imaging of the same animal at multiple time points, in contrast to histological evaluation, which requires sacrifice at predefined stage.

As noted above, radiological outcomes do not necessarily correlate with either clinical or histological results; therefore, imaging studies should not be considered the sole method for assessing treatment outcomes.

## 5 Discussion

### 5.1 Translational perspectives for autologous MSC seeded scaffold

Across the preclinical studies included in this review, autologous MSCs seeded on scaffolds consistently improved the regeneration of chondral defects, compared to empty defects or acellular scaffolds, in both small and large animal models. Most reports described more complete defect fill, greater matrix organization, stronger Safranin O and collagen II staining, and higher GAG or GAG/DNA (Zscharnack et al., 2010; Jung et al., 2009; Kilmer et al., 2020; Chen et al., 2011; Peng et al., 2019; Fan et al., 2006; Pan et al., 2015; Uematsu et al., 2005; Wege et al., 2010; Løken et al., 2008; Jia et al., 2015; Wei et al., 2015). These findings were replicated across multiple species and joint locations, indicated that MSC-scaffold constructs can be broadly implemented to enhance early stages of cartilage regeneration. Nonetheless, the regenerated tissue rarely achieved full equivalence to native hyaline cartilage and some discrepancies were reported. Wakitani S., et al. observed that the repaired cartilage was thinner at 24 weeks compared with 12 weeks, and that metachromasia was absent in some areas of the experimental group (Wakitani and Yamamoto, 2002). Similarly, Oshima Y., et al. showcased evidence that transplanted MSCs were no longer detectable at 24 weeks in the implantation site, implying a predominantly trophic rather than structural contribution (Oshima et al., 2009). One possible explanation offered was that the cells had already differentiated into chondrogenic lineages and migrated into the surrounding tissue (Oshima et al., 2009). Additionally, Wei et al. (2015) concluded that cartilage regeneration remained unsatisfactory compared to healthy tissue, while Yamasaki S., et al. (Matsumoto et al., 2010) stated that the quality of the repaired tissue was inferior to the neighboring healthy tissue. Interestingly, it was also highlighted that proper repair of underlying subchondral bone is crucial for enhancing cartilage regeneration (Zhou et al., 2006). This concept has not been deeply analyzed and even has been omitted in the majority of studies reviewed, leaving room for further research. In addition, methodological heterogeneity, inconsistent reporting of MSC source, passage, seeding density, scaffold architecture, defect size and duration of follow up, limited the ability to compare outcomes across studies or identify optimal parameters (Abdul Rahman et al., 2015; Filardo et al., 2013; Yamasaki et al., 2014; Zscharnack et al., 2010; Jung et al., 2009; Chen et al., 2011; Peng et al., 2019; Fan et al., 2006; Pan et al., 2015; Wege et al., 2010; Løken et al., 2008; Jia et al., 2015; Veronesi et al., 2013).

However, when viewed together, these preclinical data provide a foundation for translation. The repeated observation that MSC-scaffold constructs improve early defect fill and matrix quality suggest that this strategy could be integrated into existing clinical workflows similarly to matrix-assisted cartilage repair procedures. The main advantages include biological safety and immunocompatibility, as autologous sourcing minimizes the risk of immune response. Specific scaffold chemistries and architectures can be selected to accommodate a variety of defects and their anatomical locations. Many of the biomaterials used experimentally (e.g., collagen, HA and PLGA-based scaffolds) already have a clinical track record in orthopedics, making them attractive candidates for stepwise translation (Chen et al., 2011; Uematsu et al., 2005; Kayakabe et al., 2006; Løken et al., 2008).

Also, autologous BMMSCs are readily harvested and expanded using established protocols (Jung et al., 2009; Chen et al., 2011; Caminal et al., 2016; Uematsu et al., 2005; Kayakabe et al., 2006; Løken et al., 2008). On the other hand, the risks and limitations are also consistent across the analyzed studies. First, cartilage quality can be variable, with a risk of fibrocartilage formation; outcomes depend strongly on scaffold architecture and the biological environment, and there is no consensus on the optimal type or number of implanted cells, which complicates standardization (Zscharnack et al., 2010; Peng et al., 2019; Jia et al., 2015; Wei et al., 2015; Veronesi et al., 2013). Standardizing MSC characterization, dosing, seeding density, and scaffold architecture could enhance clinical translation and ensure reproducibility (Filardo et al., 2013; Yamasaki et al., 2014; Jung et al., 2009; Lv and Yu, 2015; Pan et al., 2015; De Bari et al., 2001). Also, more large-animal studies of autologous MSCs on clinically relevant scaffold with >12 months histology, MRI and mechanical testing should be done to confirm durability and integration (Jung et al., 2009; Caminal et al., 2016; Jia et al., 2015; Liang et al., 2018). It is evident that the relationship between factors such as cell dosage, scaffold type, cell passage, and the type of MSCs remains unclear and requires further standardization in order to implement these procedures clinically.

There have been some clinical attempts for such procedures in humans. Akgun et al. compared matrix-induced autologous MSC implantation with matrix-induced autologous chondrocyte implantation and observed that, in 14 patients followed for 24 months, both treatment groups showed clinical improvement; however, the MSC group demonstrated superior functional outcomes, higher MRI scores, and no cases of graft failure (Akgun et al., 2015). Also, in an observational cohort of 72 patients, MSC implantation demonstrated outcomes similar to those of ACI at the 24-month follow-up, with the added advantage of requiring one knee surgery less (Nejadnik et al., 2010).

Interpretation of the preclinical evidence presented here must account for potential sources of systematic bias. Many studies provided limited information on key methodological safeguards, such as randomization, blinding of outcome assessment, and the use of appropriate control groups. This is particularly critical in studies relying on semi-quantitative evaluations (e.g., histological or radiological scoring), where subjective expectations of the evaluator may influence the results. Furthermore, substantial heterogeneity in animal species, sample sizes, defect characteristics, intervention and scaffold types, dosing protocols, rehabilitation regimens, and follow-up durations complicates direct comparisons and likely contributes to variability in reported outcomes. Notably, studies reporting negative or null results are rarely published, creating a potential publication bias that may overestimate the apparent efficacy of interventions. Collectively, these limitations reduce confidence in the robustness and long-term durability of reported effects. They underscore the urgent need for rigorous, standardized, and transparent reporting in preclinical research to enable meaningful synthesis of evidence and to better inform the design of subsequent translational and clinical studies (Hooijmans et al., 2014; Percie du Sert et al., 2020).

Accordingly, selecting an appropriate animal model is a key determinant of both the interpretability and the translational relevance of preclinical cartilage repair studies. Animal models differ in their strengths and limitations. Compared with small animals, large-animal models (e.g., dogs, minipigs, sheep, goats, and horses) more closely resemble humans with respect to joint anatomy and biomechanics, cartilage and subchondral bone structure, and biological responses to injury. They also allow the use of standard clinical imaging, arthroscopic procedures, and more human-relevant postoperative management, thereby improving translational relevance. In addition, large animals develop post-traumatic and age-related knee osteoarthritis through mechanisms similar to humans, including meniscal injury, osteochondrosis, and degenerative change (Oláh et al., 2021). Nevertheless, species-specific differences can substantially influence preclinical outcomes and should be carefully weighed when selecting a model. This requires familiarity not only with macroscopic and microscopic anatomy and pathology, but also with each species’ gait pattern and joint kinematics. Because no available animal model fully replicates the characteristics of the human knee, model selection should be guided by the specific research question and a clear appraisal of the trade-offs that are acceptable for the planned study (Moran et al., 2016; Oláh et al., 2021).

## 6 Future

The immediate translational goal is to develop, simple and economically viable strategies that stimulate joint cartilage regeneration. MSC-scaffold construct clinical usage depends on standardizing MSC-scaffold protocols and embedding them into pragmatic clinical trials that compare autologous MSC-seeded constructs with established options such as microfracture, marrow stimulation, or autologous chondrocyte implantation (Bornes et al., 2014; Filardo et al., 2013; Katayama et al., 2004; Nejadnik et al., 2010). Apart from that, parallel work should focus on perioperative workflows, optimizing the procedure, refining rehabilitation protocols, and incorporating health-economic evaluations, to ensure that any benefits in cartilage quality translate into long-term OA symptoms relief and cost-effective care.

Looking ahead, cartilage regeneration is likely to move toward fully personalized, advanced tissue-engineering strategies, building on current MSC-based approaches rather than replacing them. Rapid progression in 3D printing and bioprinting enables the fabrication of patient-specific chondral constructs with zonal organization, specific pore architecture and mechanical properties resembling those of native tissue (Pan et al., 2015; Rajagopal et al., 2020). Recent reviews describe 3D bioprinting of hyaline articular cartilage using bioink that combine biopolymers, hydrogels and living cells, bringing clinically relevant, stratified grafts within reach (Vo et al., 2023). Advances in bioreactor technology, which already allow precise control of physicochemical conditions and mechanical loading *in vitro*, are expected to support routine precondition of patient-derived constructs so that implants enter the joint with optimized biochemical and biomechanical properties (Mauck et al., 2000; Martin et al., 2004).

Concurrently, cell-free yet cell-inspired therapies are gaining attention. Exosomes and other extracellular vesicles derived from MSCs or chondrocytes can recapitulate many of the paracrine functions of transplanted cells, while circumventing challenges related to cell survival, engraftment, and hypertrophy (Swami et al., 2024). Emerging evidence suggests that these vesicles modulate inflammation, extracellular matrix turnover, and subchondral bone remodeling in models of OA and cartilage injury, and that engineered vesicles can be tailored to address disease- or patient-specific needs (Yang et al., 2025). Within this framework, current MSC-scaffold strategies may evolve into platforms capable of delivering both living cells and bioactive vesicles, or eventually give rise to entirely acellular implants.

In summary, the field is moving toward a future in which a single, highly personalized procedure could restore a focal cartilage defect with mechanically competent, biologically integrated tissue engineered from the patient’s own cells and biomaterials. Realizing this vision will require advances in biomaterials, bioreactors, bioprinting, and vesicle engineering, alongside robust regulatory and ethical frameworks to ensure safety, standardization, and equitable access. Successfully addressing these challenges could enable MSC-based and next-generation tissue engineering strategies to transform the management of cartilage injury and OA, reducing disability, delaying or preventing arthroplasty, and improving long-term joint health at the population level.
