# Supplementary material for: Models of cartilage repair with autologous mesenchymal stem cells seeded on scaffolds: a systematic narrative review
Source: Front Bioeng Biotechnol. 2026 Mar 3;14:1762579. doi: 10.3389/fbioe.2026.1762579 (PMC13040362; doi:10.3389/fbioe.2026.1762579)
Supplement: Supplementary file 1 [file Supplementaryfile1.docx]

**Supplementary material S1.**

***Pubmed (334 results)***

Search: **((cartilage regeneration) or (osteochondral defect)) AND ((autologous bone marrow cells))** ((("cartilage"[MeSH Terms] OR "cartilage"[All Fields] OR "cartilages"[All Fields] OR "cartilage s"[All Fields] OR "cartilageous"[All Fields]) AND ("regenerability"[All Fields] OR "regenerable"[All Fields] OR "regenerant"[All Fields] OR "regenerants"[All Fields] OR "regenerate"[All Fields] OR "regenerated"[All Fields] OR "regenerates"[All Fields] OR "regenerating"[All Fields] OR "regeneration"[MeSH Terms] OR "regeneration"[All Fields] OR "regenerations"[All Fields])) OR (("osteochondral"[All Fields] OR "osteochondritis"[MeSH Terms] OR "osteochondritis"[All Fields] OR "osteochondritides"[All Fields]) AND ("abnormalities"[MeSH Subheading] OR "abnormalities"[All Fields] OR "defects"[All Fields] OR "defect"[All Fields] OR "defect s"[All Fields] OR "defected"[All Fields] OR "defective"[All Fields] OR "defectively"[All Fields] OR "defectives"[All Fields]))) AND (("autolog"[All Fields] OR "autologeous"[All Fields] OR "autologic"[All Fields] OR "autological"[All Fields] OR "autologous"[All Fields] OR "autologously"[All Fields]) AND ("bone marrow cells"[MeSH Terms] OR ("bone"[All Fields] AND "marrow"[All Fields] AND "cells"[All Fields]) OR "bone marrow cells"[All Fields]))

**Translations**

**cartilage:** "cartilage"[MeSH Terms] OR "cartilage"[All Fields] OR "cartilages"[All Fields] OR "cartilage's"[All Fields] OR "cartilageous"[All Fields]

**regeneration:** "regenerability"[All Fields] OR "regenerable"[All Fields] OR "regenerant"[All Fields] OR "regenerants"[All Fields] OR "regenerate"[All Fields] OR "regenerated"[All Fields] OR "regenerates"[All Fields] OR "regenerating"[All Fields] OR "regeneration"[MeSH Terms] OR "regeneration"[All Fields] OR "regenerations"[All Fields]

**osteochondral:** "osteochondral"[All Fields] OR "osteochondritis"[MeSH Terms] OR "osteochondritis"[All Fields] OR "osteochondritides"[All Fields]

**defect:** "abnormalities"[Subheading] OR "abnormalities"[All Fields] OR "defects"[All Fields] OR "defect"[All Fields] OR "defect's"[All Fields] OR "defected"[All Fields] OR "defective"[All Fields] OR "defectively"[All Fields] OR "defectives"[All Fields]

**autologous:** "autolog"[All Fields] OR "autologeous"[All Fields] OR "autologic"[All Fields] OR "autological"[All Fields] OR "autologous"[All Fields] OR "autologously"[All Fields]

**bone marrow cells:** "bone marrow cells"[MeSH Terms] OR ("bone"[All Fields] AND "marrow"[All Fields] AND "cells"[All Fields]) OR "bone marrow cells"[All Fields]

***Scopus ( 535 results)***

((cartilage AND regeneration) OR (osteochondral AND defect)) AND ((autologous AND bone AND marrow AND cells))

***Web of science ( 869 results)***

ALL=((((cartilage regeneration) or (osteochondral defect) ) AND ((autologous bone marrow cells))))

***Embase ( 698 results)***

('cartilage regeneration'/exp OR 'cartilage regeneration' OR (('cartilage'/exp OR cartilage) AND ('regeneration'/exp OR regeneration)) OR 'osteochondral defect'/exp OR 'osteochondral defect' OR (osteochondral AND defect)) AND ('autologous bone marrow cells' OR (autologous AND ('bone'/exp OR bone) AND ('marrow'/exp OR marrow) AND ('cells'/exp OR cells)))
